# Sensitive detection of pathological seeds of α-synuclein, tau and prion protein on solid surfaces

**DOI:** 10.1371/journal.ppat.1012175

**Published:** 2024-04-19

**Authors:** Christina D. Orrú, Bradley R. Groveman, Andrew G. Hughson, Tomás Barrio, Kachi Isiofia, Brent Race, Natalia C. Ferreira, Pierluigi Gambetti, David A. Schneider, Kentaro Masujin, Kohtaro Miyazawa, Bernardino Ghetti, Gianluigi Zanusso, Byron Caughey

**Affiliations:** 1 Laboratory of Neurological Infections and Immunity (LNII), Rocky Mountain Laboratories, Division of Intramural Research, National Institute of Allergy and Infectious Diseases, National Institutes of Health, Hamilton, Montana, United States of America; 2 UMR INRAE ENVT 1225, Interactions Hôtes-Agents Pathogènes, École Nationale Vétérinaire de Toulouse, France; 3 Department of Pathology, Case Western Reserve University, Cleveland, Ohio, United States of America; 4 Animal Disease Research Unit, USDA-ARS, Pullman, Washington, United States of America; 5 National Institute of Animal Health (NIAH), National Agriculture and Food Research Organization (NARO), Tsukuba, Ibaraki, Japan; 6 Department of Pathology and Laboratory Medicine, Indiana University, Indianapolis, Indiana, United States of America; 7 Department of Neurosciences, Biomedicine and Movement Sciences, University of Verona, Verona, Italy; Colorado State University College of Veterinary Medicine and Biomedical Sciences, UNITED STATES

## Abstract

Prions or prion-like aggregates such as those composed of PrP, α-synuclein, and tau are key features of proteinopathies such as prion, Parkinson’s and Alzheimer’s diseases, respectively. Their presence on solid surfaces may be biohazardous under some circumstances. PrP prions bound to solids are detectable by ultrasensitive real-time quaking-induced conversion (RT-QuIC) assays if the solids can be immersed in assay wells or the prions transferred to pads. Here we show that prion-like seeds can remain detectable on steel wires for at least a year, or even after enzymatic cleaning and sterilization. We also show that contamination of larger objects with pathological seeds of α-synuclein, tau, and PrP can be detected by simply assaying a sampling medium that has been transiently applied to the surface. Human α-synuclein seeds in dementia with Lewy bodies brain tissue were detected by α-synuclein RT-QuIC after drying of tissue dilutions with concentrations as low as 10^−6^ onto stainless steel. Tau RT-QuIC detected tau seeding activity on steel exposed to Alzheimer’s disease brain tissue diluted as much as a billion fold. Prion RT-QuIC assays detected seeding activity on plates exposed to brain dilutions as extreme as 10^−5^–10^−8^ from prion-affected humans, sheep, cattle and cervids. Sampling medium collected from surgical instruments used in necropsies of sporadic Creutzfeldt-Jakob disease-infected transgenic mice was positive down to 10^−6^ dilution. Sensitivity for prion detection was not sacrificed by omitting the recombinant PrP substrate from the sampling medium during its application to a surface and subsequent storage as long as the substrate was added prior to performing the assay reaction. Our findings demonstrate practical prototypic surface RT-QuIC protocols for the highly sensitive detection of pathologic seeds of α-synuclein, tau, and PrP on solid objects.

## Author summary

Prions and prion-like protein aggregates associated with neurodegenerative diseases such as Creutzfeldt-Jakob disease, Alzheimer’s disease, and dementia with Lewy bodies may persist in the environment or on medical instruments and in some cases be transmissible. Sensitive detection of these self-propagating aggregates, or seeds, on solid surfaces, machinery, tools, and surgical instruments might help prevent fomite-borne disease dissemination. Here we describe a novel RT-QuIC (sfRT-QuIC) to detect a variety of pathological brain-derived prion, α-synuclein, or tau seeds on stainless steel and acrylic surfaces. sfRT-QuIC testing showed that α-synuclein and tau seeds remained active on surgical forceps and scissors, even after routine instrument sterilization. Furthermore, we found that pathological protein seeds eluted from surfaces could be collected and stored for transport or delayed testing. sfRT-QuIC is a simple and effective, high throughput assay for sensitive detection of pathological protein seed contamination of common surfaces.

## Introduction

Multiple neurodegenerative diseases involve the accumulation of aggregates of specific proteins, often in the form of self-propagating and sometimes transmissible cross-β amyloid fibrils [1–5]. The experimental transmissibility of several of these pathologic protein aggregates has raised concerns about potential natural or iatrogenic routes of transmission of such proteinopathies [3,6–8]. Such transmissions have been most thoroughly documented for the mammalian PrP-based prion diseases, including Creutzfeldt-Jakob disease (CJD) and GSS [9] in humans, chronic wasting disease (CWD) in cervids, scrapie in sheep and goats, and bovine spongiform encephalopathy (BSE) [10]. The molecular pathogenesis of PrP prion diseases is based on the conversion of the hosts’ normal monomeric prion protein (PrP^C^) into a refolded, aggregated, and infectious state (generically called PrP^Sc^) in which, for the prion structures that have been solved to date, PrP molecules become stacked via parallel in-register intermolecular β-sheets (PIRIBS) into amyloid fibrils [11–13]. Relative to most pathogens, prions are unusually persistent in the environment [14–19] and more difficult to inactivate (e.g. [20,21]). Exposure to contaminated surgical instruments has initiated fatal human prion disease cases [22–24]. Scrapie and CWD have been transmitted via contamination of environmental surfaces (e.g., [15,25,26]). Another type of prion, i.e., the α-synuclein (α-syn)-based prions of multiple system atrophy (MSA), have been experimentally transmitted via suture wires exposed to human MSA brain homogenate [8]. The assessment and minimization of risks of iatrogenic or environmentally mediated proteinopathy transmission would be aided by the availability of practical methods for detecting contamination of various surfaces with prion or prion-like protein aggregates.

The development of prion seed amplification assays (SAAs) [27–29] has enabled the ultrasensitive detection of various types of prions and prion-like protein aggregates of PrP, α-syn, tau, and Aβ to the attogram to low femtogram range [29–32]. In current real-time quaking-induced conversion (RT-QuIC) SAAs, self-propagating protein aggregates are detected based on their ability to seed the conversion of recombinant PrP, α-Syn or tau monomers into amyloid fibrils that can then be detected using an amyloid-sensitive fluorescent dye, thioflavin T (ThT) [29]. RT-QuIC assays have been designed for high throughput analysis in 96 or 384-well plates and can provide rough quantitation through end-point titration analysis [29] or, under more carefully controlled conditions, comparison of reaction kinetics at single dilutions [33,34]. SAAs can detect PrP prion seeds bound to solid surfaces such as steel wires when the material is small enough to be added to the reaction microtube or well [18,35–39]. However, a limitation of most existing assays is the inability to detect prions that are bound to materials that interfere with the assay or will not fit into an assay well. Two recent studies reported detection of CWD prions from flat surfaces by sampling with foam swabs that are sonicated to elute prion seeds that are then concentrated and analyzed by RT-QuIC and PMCA [19,40]. Here, we demonstrate a more direct approach for detecting a broad range of pathologic PrP, α-Syn and tau prions or prion-like aggregates on large surfaces by simply exposing a sampling media to a surface (e.g., a surgical instrument) and then transferring the solution into an RT-QuIC assay plate for analysis. These prototypic surface RT-QuIC (sfRT-QuIC) assays provide methods for assessing contamination of medical instruments or other solid fomites that may be sources of proteinopathy infections in clinical, agricultural, and wildlife settings.

## Results

### sfRT-QuIC detection of 263K prion seeds 1 year after contamination on stainless steel

**Fig 1:**
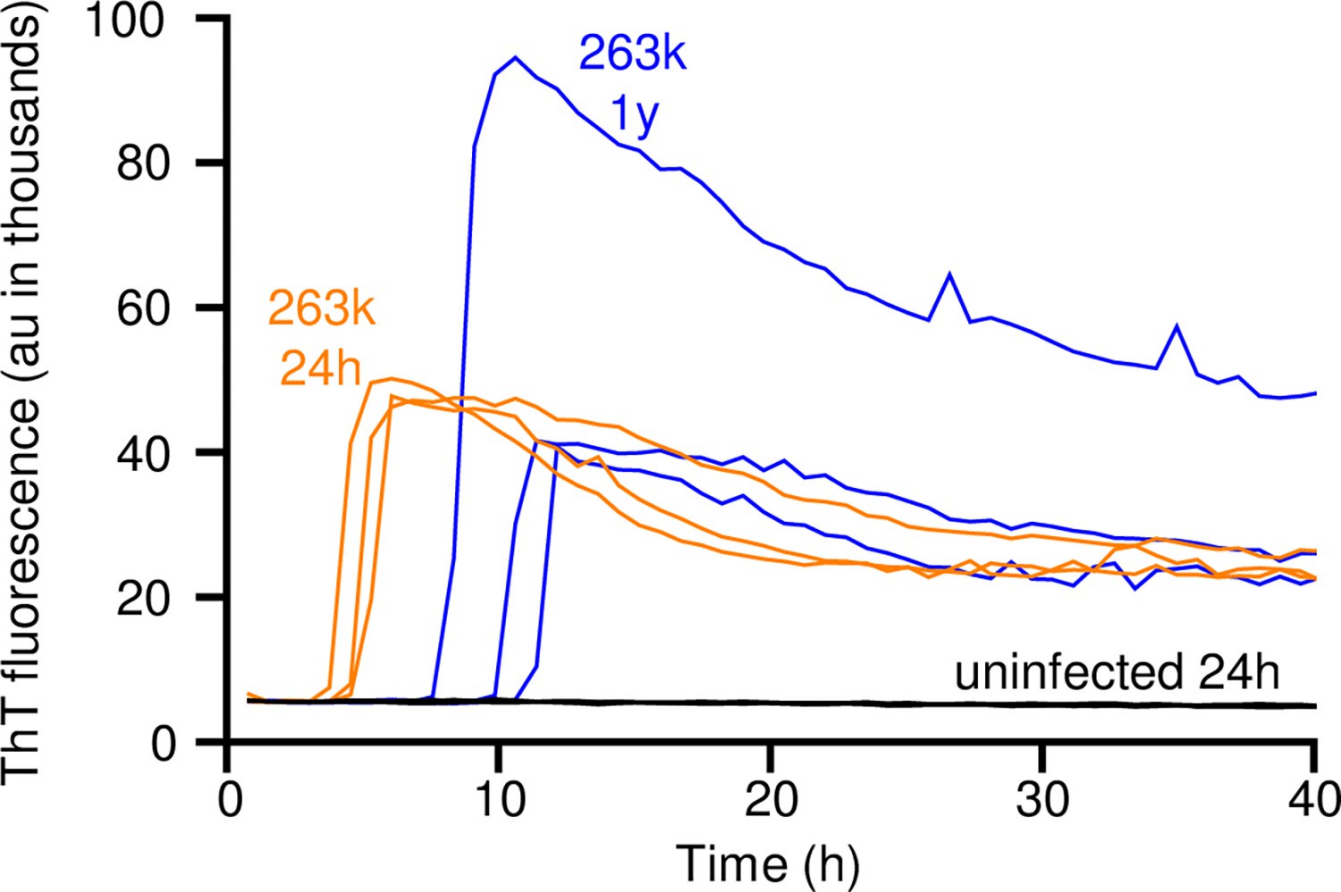
RT-QuIC detection of 263K prion seeding activity on stainless steel wires after 1 year. Stainless-steel wires were coated with 10^−3^ brain equivalents of 263K or uninfected brain tissue, dried, and inserted in individual wells for RT-QuIC analysis (one wire per well). One set of wires was tested after drying for 24 h (263K, orange; uninfected, black; n = 3); another set (263K only) was tested after 1 y of storage at room temperature (blue; n = 3). ThT fluorescence for each reaction is plotted vs. time.

Although we and others have previously detected prion seeding activity on stainless steel wires using SAAs such as RT-QuIC [35], we first sought evidence of whether seeding activity can persist long term on stainless steel. We dipped steel wires into a 10^−3^ (w/v) dilution of brain tissue (homogenized) from a hamster infected with the 263K scrapie strain, rinsed them in water, and stored them for 1 y prior to RT-QuIC testing in comparison to freshly contaminated wires. For testing, the wires were added directly to wells of an RT-QuIC test plate and left there for the duration of the assay reaction. Strong positive RT-QuIC responses were obtained in both the freshly exposed and 1 y samples, but not with wires exposed to uninfected hamster brain homogenate (Fig 1). Slightly slower reaction kinetics were observed in the 1 y sample suggesting that long terms storage may result in a modest reduction of seeding activity.

### Detecting prion seeds dried onto flat stainless-steel or acrylic surfaces

**Fig 2:**
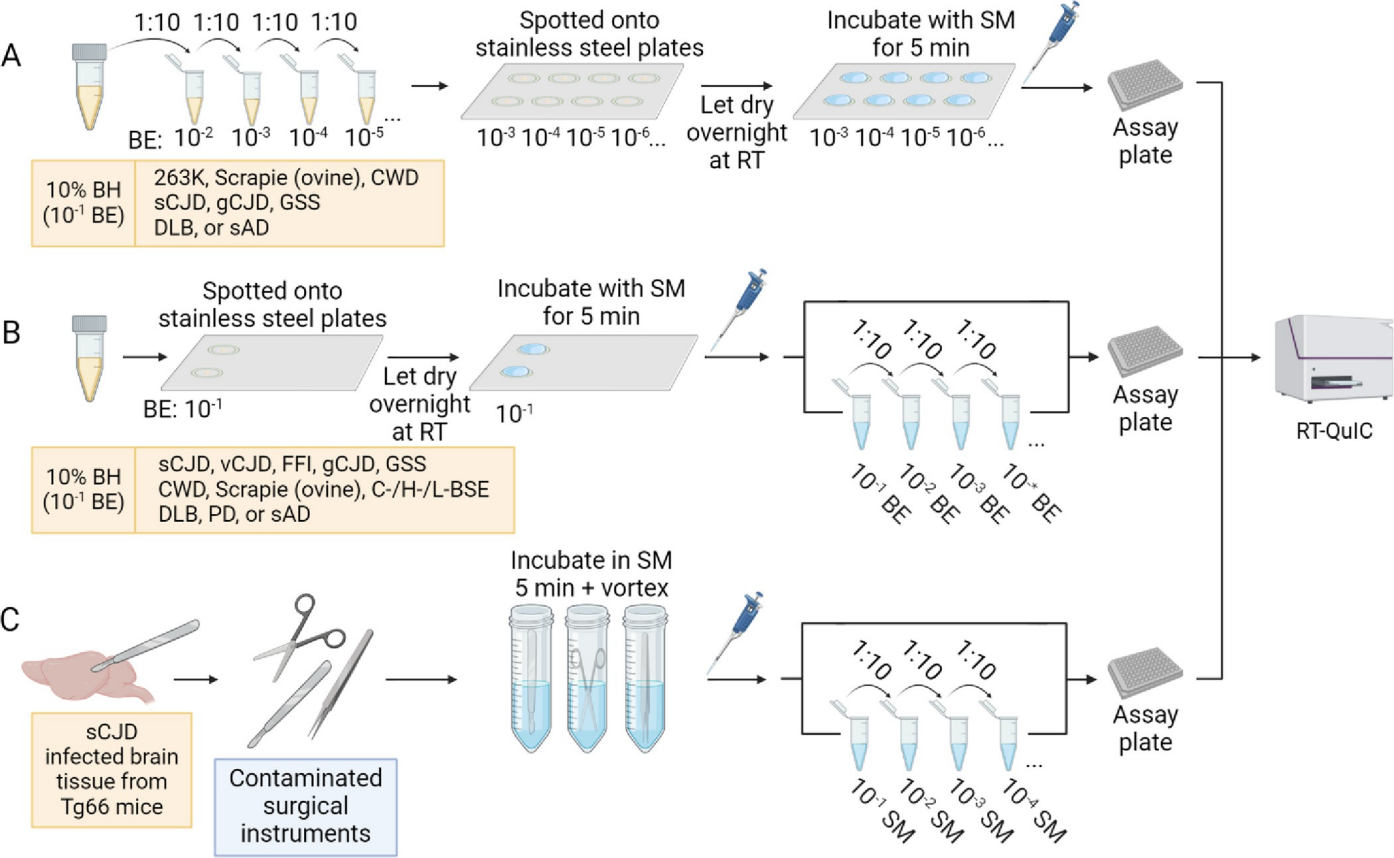
sfRT-QuIC experimental design schematic. **Panel A.** Spotting of surfaces with serially diluted brain homogenate (BH, yellow liquid; BE: brain equivalents) followed by sfRT-QuIC testing of sampling medium (SM; blue liquid). **Panel B**. Spotting of surfaces with 10% BH followed by serial dilutions of the SM for sfRT-QuIC. **Panel C.** sfRT-QuIC of surgical instruments contaminated with CJD-infected humanized mouse brain tissue: sampling procedure and serial dilutions of the SM for sfRT-QuIC. Created with BioRender.com.

**Fig 3:**
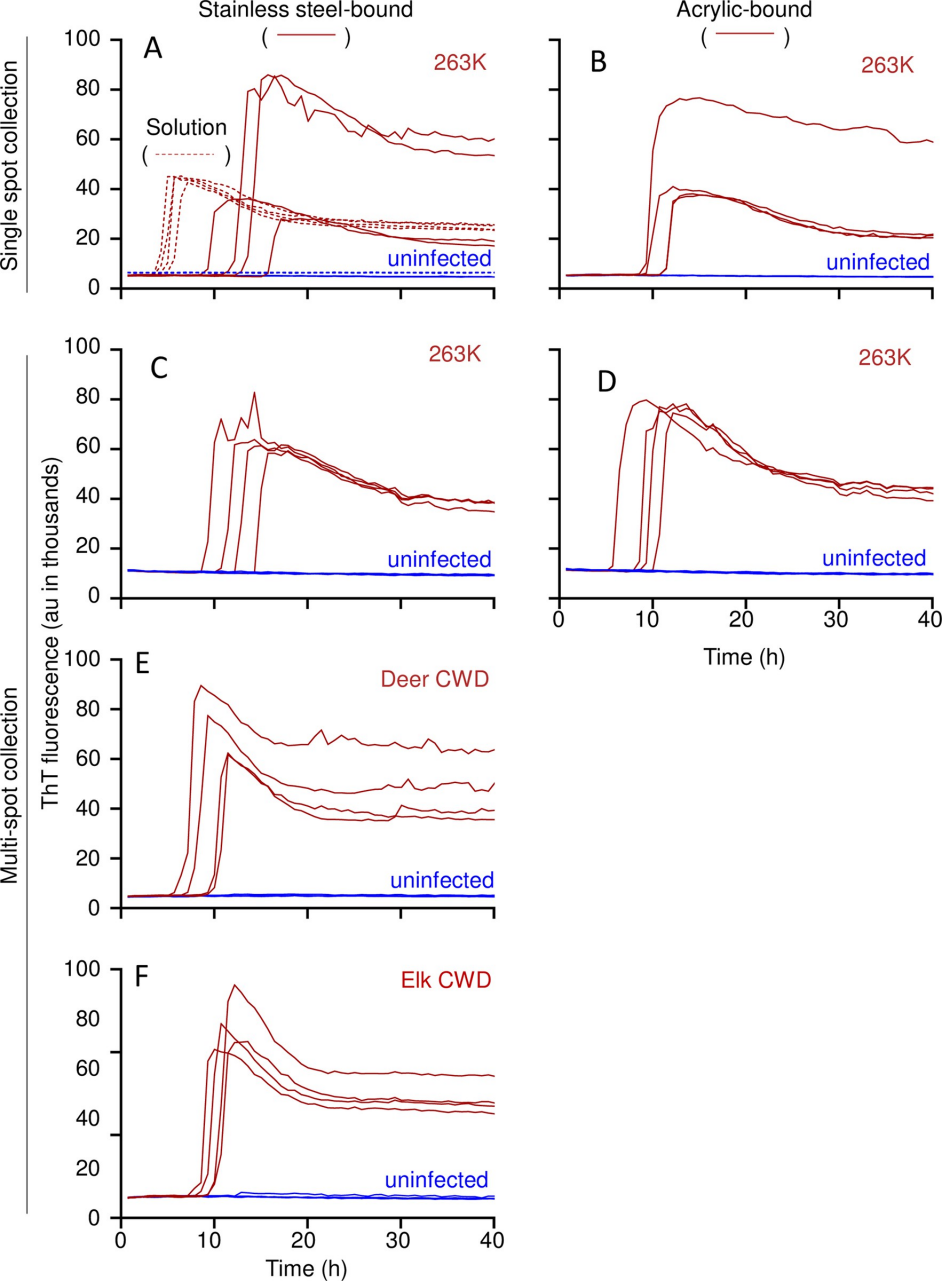
sfRT-QuIC of stainless steel- or acrylic-bound 263K, CWD, or uninfected brain homogenates. **Panel A-B.** sfRT-QuIC of SM collected from a single spot from 263K (red, full line) or uninfected (blue, full line) BH (10^−4^ dilution) dried onto stainless steel (A) or acrylic (B) surfaces or in solution (A; red and blue dashed lines). **Panel C-D.** sfRT-QuIC of a 2 mL SM collection from 8 263K (red) or uninfected (blue) spottings placed in a random pattern within a test circle on stainless steel (**C**) or acrylic (**D**). Each collection was tested in quadruplicate. **Panel E-F.** sfRT-QuIC of SM collected as in **C,D** from spottings of CWD (red) or uninfected (blue) BH (10^−4^ dilutions) from either deer (E) or elk (F). The recombinant PrP substrates used are described in Methods.

We then tested if prion contamination can be detected on larger flat steel or acrylic surfaces by transiently exposing them to RT-QuIC sampling medium (SM: RT-QuIC reaction mix; see methods and Fig 2A). In pilot experiments (Fig 3A), 4 aliquots of 263K or normal hamster brain homogenates (2 μL of 10^−4^ solid brain tissue equivalent dilution) were spotted onto stainless steel plates or acrylic sheets and allowed to air dry 16–24 h in a biosafety hood with active laminar flow. Sampling medium (100 μl) was delivered as a bead of liquid over each spot and allowed to sit for 5 min at room temperature. The SM was pipetted up and down 3 times and ~95 μl was recovered, transferred to the well of a 96-well plate, and subjected to the remainder of the RT-QuIC protocol as above. Tests of all 4 replicate collections of the scrapie brain on either stainless-steel (Fig 3A) or acrylic (Fig 3B) were positive in the RT-QuIC assay, while those of the uninfected brain were negative. Similar results were obtained from a larger-volume protocol in which two 40 μl spots of 10^−3^ dilutions of 263K, deer CWD, or elk CWD brain homogenates were placed within a ~ 3 cm circle on stainless-steel (Fig 3C,3E and 3F) or acrylic (Fig 3D) and sampled with 2 ml SM (Fig 3C–3F).

### Detection of disease-associated α-Syn, tau, and prion seeds spotted on stainless steel surfaces: sfRT-QuIC

**Fig 4:**
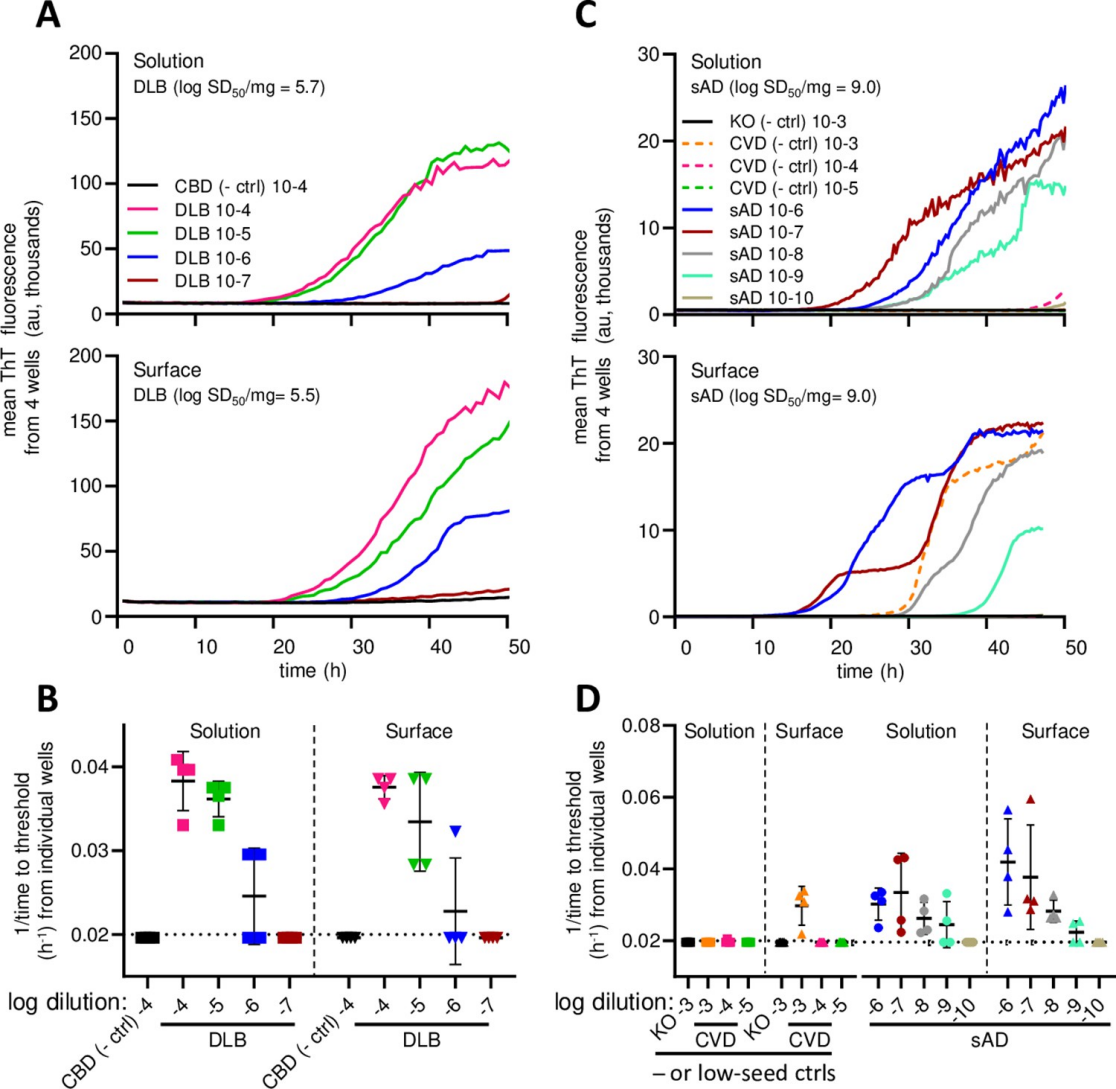
sfRT-QuIC detection of α-synuclein and tau seeding activity from brain homogenates. **Panel A.** Designated dilutions of DLB or corticobasal degeneration (CBD, a non-synucleinopathy control) tested by solution or sfRT-QuIC. Curves are averages of 4 replicate reactions. Y-axis indicates mean ThT fluorescence from replicate wells and x-axis assay duration (h). **Panel B** 1/time to fluorescence positivity threshold for each DLB and CBD reaction versus the dilution (log_10_ tissue equivalents). The dotted horizontal line represents the inverse of the time of the final measurement. The dashed vertical line separates solution vs. surface data. The mean (heavy horizontal lines) and standard deviations (vertical) are displayed for each dilution tested. **Panel C.** Designated dilutions of sAD or cerebrovascular disease (CVD; a low tau seed control) BH tested by solution and sfRT-QuIC. Curves as in **A**. **Panel D.** 1/time to threshold for each reaction seeded with brain homogenate at the designated tissue dilutions from sAD, Tau KO (a tau-negative control), or CVD (a low, but not completely negative, tau seed control) with labeling as in **B**.

Based on these initial findings, we further investigated the ability of this overall assay strategy, which we will call surface RT-QuIC, or sfRT-QuIC, to detect contamination of pathological seeds of α-Syn, tau, and PrP on stainless-steel surfaces (Fig 2). For the α-Syn and tau seed experiments, we used brain homogenates from patients with neuropathologically confirmed diagnoses of dementia with Lewy Bodies (DLB) or sporadic Alzheimer’s disease (sAD), respectively. Two μL of serial 10-fold dilutions of the brain homogenates were spotted onto stainless steel plates and allowed to air dry 16–24 h (Fig 2A). A bead of SM (100 μl) was applied to each spot and allowed to sit for 5 min. The SM was pipetted up and down 3 times and ~95 or 48 μl was recovered, transferred to a well of a 96- or 384-well plate, respectively (the latter for tau detection), and subjected to one of three of our previously described RT-QuIC protocols for either α-Syn, tau, or PrP seeds. We compared our sfRT-QuIC results to those obtained from standard solution RT-QuIC assays in which the same homogenates were pipetted directly into the reaction wells (Fig 4). Comparable end-point dilutions were obtained with both the solution and surface versions of the α-synuclein and tau RT-QuIC assays when seeded with DLB (Fig 4A and 4B) or sAD (Fig 4C and 4D) brain homogenates, respectively. Specifically, at least one positive in four replicate assay wells were obtained in both assay protocols with tissue dilutions of 10^−4^–10^−6^ (DLB) and 10^−6^–10^−9^ (sAD). Similar estimates of the measured seed concentrations (log 50% seeding dose, or log SD50, per mg original tissue) were obtained by application of the Spearman-Kärber algorithm [41]. In contrast, in negative control assays, no α-syn seeding activity was detected in either solution RT-QuIC or sfRT-QuIC using a negative control, non-synucleinopathy, brain homogenate from a patient with a primary tauopathy (cortico-basal degeneration) at 10^−4^ dilution (Fig 4A). Also, no tau seeding activity was detected in brain homogenate from a completely tau-free knockout (KO) mouse at 10^−3^ dilution (Fig 4C and 4D). However, we detected some tau seeding activity in tau sfRT-QuIC using the highest (10^−3^) concentration (only) of human brain homogenate from a patient with cerebrovascular disease (CVD), but without any known tauopathy (Fig 4D). This was not surprising because we have often detected low levels of tau seeding activity in brain tissue from CVD cases and multiple other types of cases without primary tauopathy [42–44], albeit at concentrations several orders of magnitude lower than those found in sAD brain, as seen here as well (Fig 4D).

**Fig 5:**
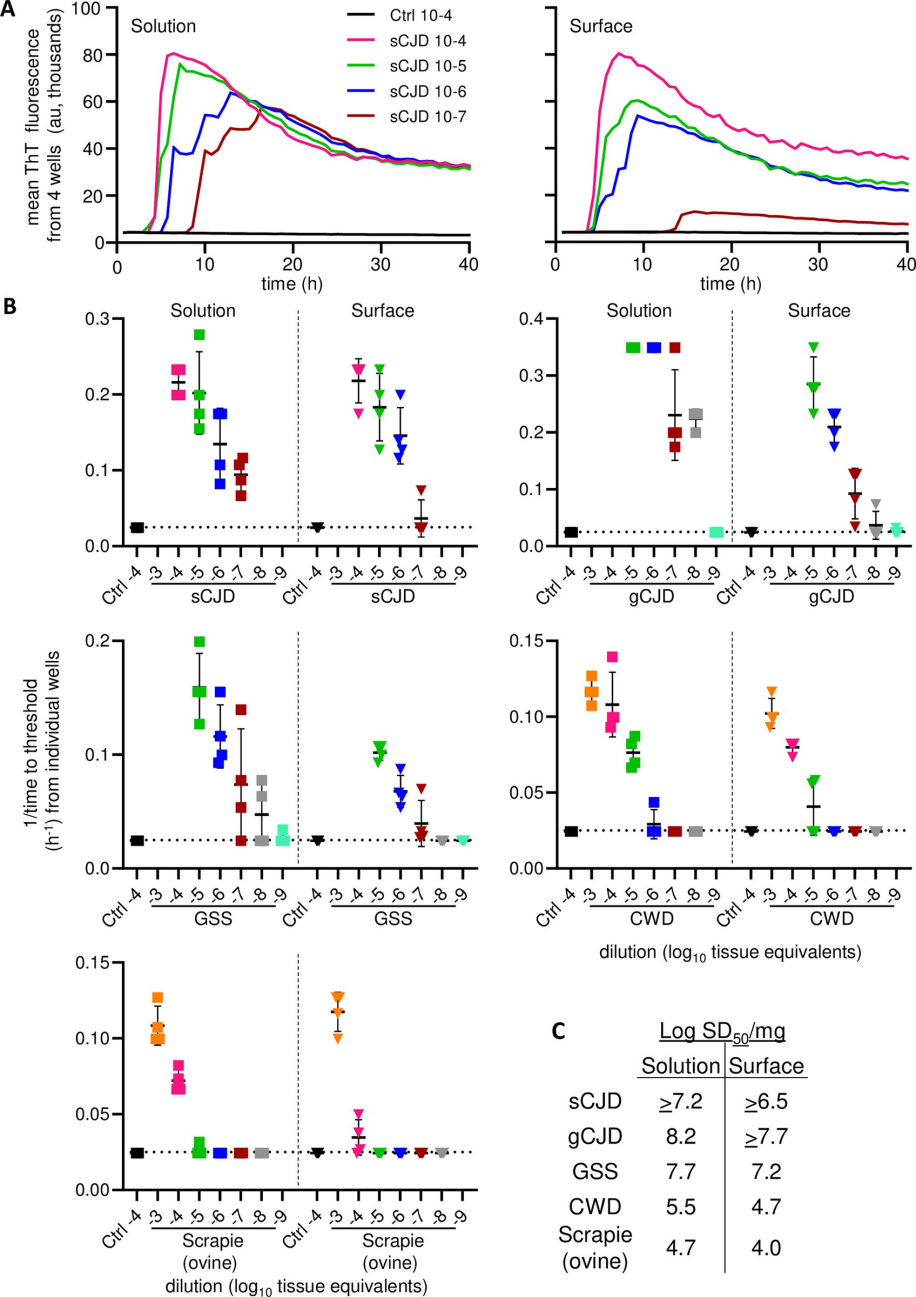
sfRT-QuIC detection of seeding activity from different strains of prion-infected brain homogenates. **Panel A.** Designated dilutions of sCJD (MM1 type) and sAD (prion negative control) BHs tested by solution (left) or sfRT-QuIC (right). Curves are show mean ThT fluorescence from quadruplicate wells versus assay duration. **Panel B.** 1/time to threshold comparisons of solution and sfRT-QuIC data from dilutions of sCJD (MM1 subtype; extracted from data shown in **A**), genetic Creutzfeldt-Jakob disease (gCJD; E200K), Gerstmann-Sträussler-Scheinker disease (GSS; P102L), ovine classical scrapie and CWD and uninfected controls (Ctrl) BHs. X-axis indicates the dilution (log_10_ tissue equivalents). **Panel C.** Table of Log SD_50_/mg original tissue values estimated using Spearman-Karber analysis of the data shown in **Panel B** as described in Methods. Comparable results were seen in analogous experiments performed with sCJD, ovine classical scrapie and CWD, except for the inclusion of 0.1% SDS in the brain homogenate diluent (data available on request).

To assess the sensitivity of sfRT-QuIC with a variety of PrP prion strains, end-point dilution analyses were performed on brain homogenates (Fig 2A) from cases of human sporadic (s) CJD (MM1 subtype), genetic (g) CJD (E200K mutant), Gerstmann-Sträussler-Scheinker disease (GSS; P102L mutant); ovine classical scrapie (ARQ/ARQ PrP genotype), and deer CWD (Fig 5). Although sfRT-QuIC sensitivities for GSS, CWD and scrapie brain samples were lower compared to the solution-based RT-QuIC, they differed by less than 10-fold (Fig 5C). Whereas these various prion-infected brain tissues gave positive sfRT-QuIC assays out to dilutions of 10^−5^ to 10^−8^, depending upon strain, negative control (uninfected) brain tissues were negative at 10^−4^. Altogether, these results indicated that sfRT-QuIC is nearly as sensitive as direct solution RT-QuIC in detecting a variety of prion strains in brain homogenates.

**Fig 6:**
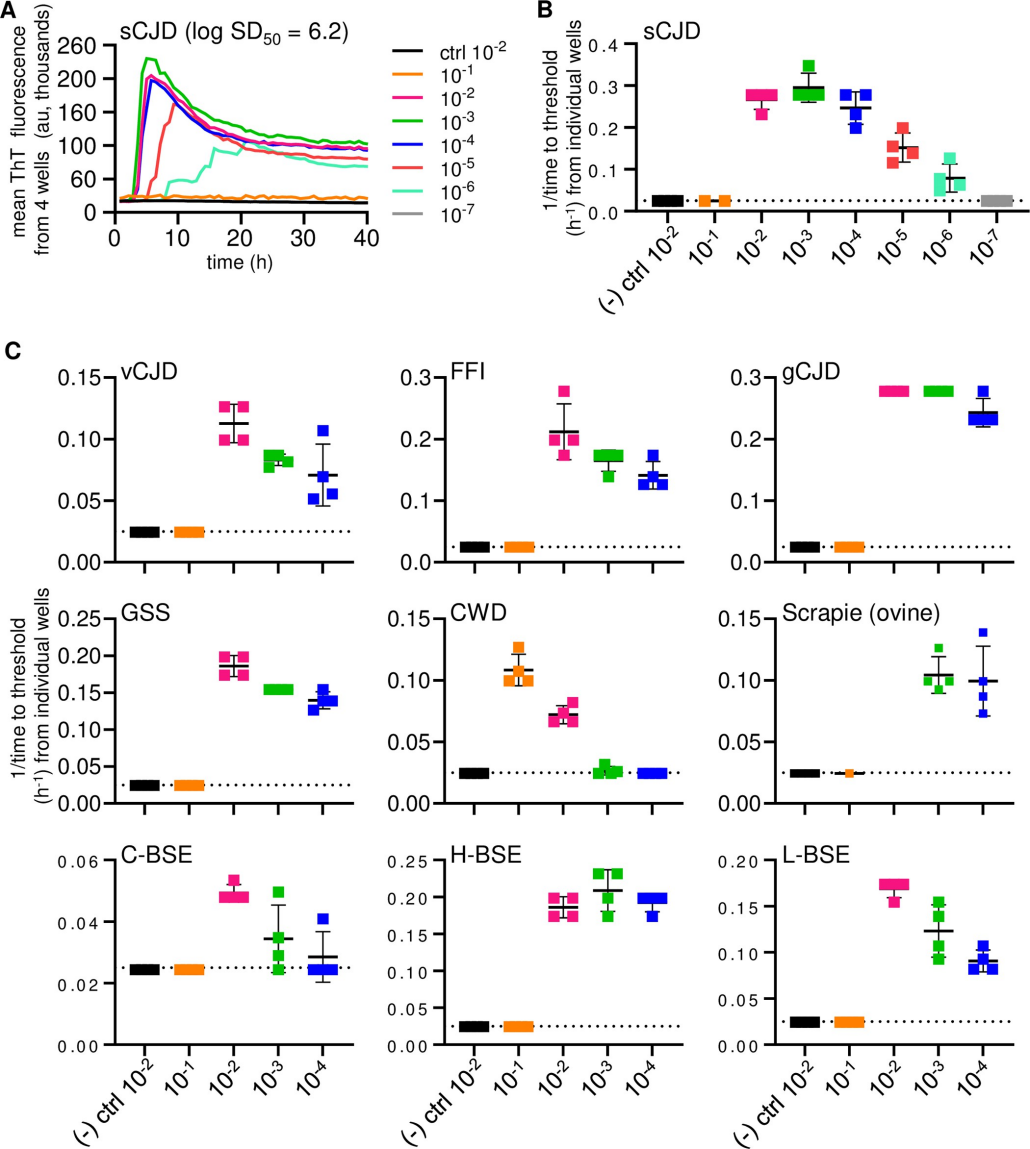
sfRT-QuIC of stainless-steel bound brain homogenate derived human, cervid, ovine, and bovine prions. **Panel A**. sfRT-QuIC of diluted SM collections from sCJD (MM1 type) or Alzheimer’s disease (as a prion-negative control) spots on a stainless-steel plate: average ThT fluorescence of 4 replicate wells versus reaction time. **Panel B.** sfRT-QuIC results from panel A represented as 1/time to threshold for each reaction seeded with the designated dilutions (log_10_ tissue equivalents; x-axis) as described initially in legend of Fig 4B and 4D. **Panel C.** Analogous data from plates contaminated with 10% BH from humans with variant Creutzfeldt-Jakob disease (vCJD), fatal familial insomnia (FFI), genetic Creutzfeldt-Jakob disease (gCJD; E200K), and Gerstmann-Sträussler-Scheinker disease (GSS; P102L); cervids with CWD; sheep with scrapie; cows with classical (C-), high- (H-) and low- (L-) BSE; and species-matched uninfected controls.

To quantify the recovery of prion seeding activity from a surface (Fig 2B), SM was applied to 2 μL of 10% human sCJD brain homogenate spots dried overnight onto stainless steel and end-point dilutions of the respective SMs were performed. No positive reactions were obtained using uninfected control brain homogenates. Neat sCJD SM were also negative presumably because, as previously observed, excessive brain tissue matrix can inhibit RT-QuIC assays [45]. However, positive reactions were obtained from further dilutions out to 10^−6^ (Fig 6A–6B), which, when considered in terms of the brain equivalents delivered to the stainless steel, corresponded to recoveries of a majority of the seeding activity initially applied to the stainless-steel. Assays were also done with other prion-infected brain homogenates assayed out to 10^−4^, namely human variant CJD, fatal familial insomnia D178N, gCJD E200K, and GSS P102L; deer CWD; sheep scrapie; bovine classical C-BSE, and atypical H- and L-types of BSE) (Fig 6C). For all but the C-BSE and CWD samples, which were known to have low seeding activity, each of these dilutions were RT-QuIC-positive in all quadruplicate wells. Thus, even after drying of brain homogenates onto stainless-steel surfaces overnight, substantial proportions of prion seeding activity can be recovered in a simple SM elution step.

### Prion detection on surgically contaminated necropsy instruments

**Fig 7:**
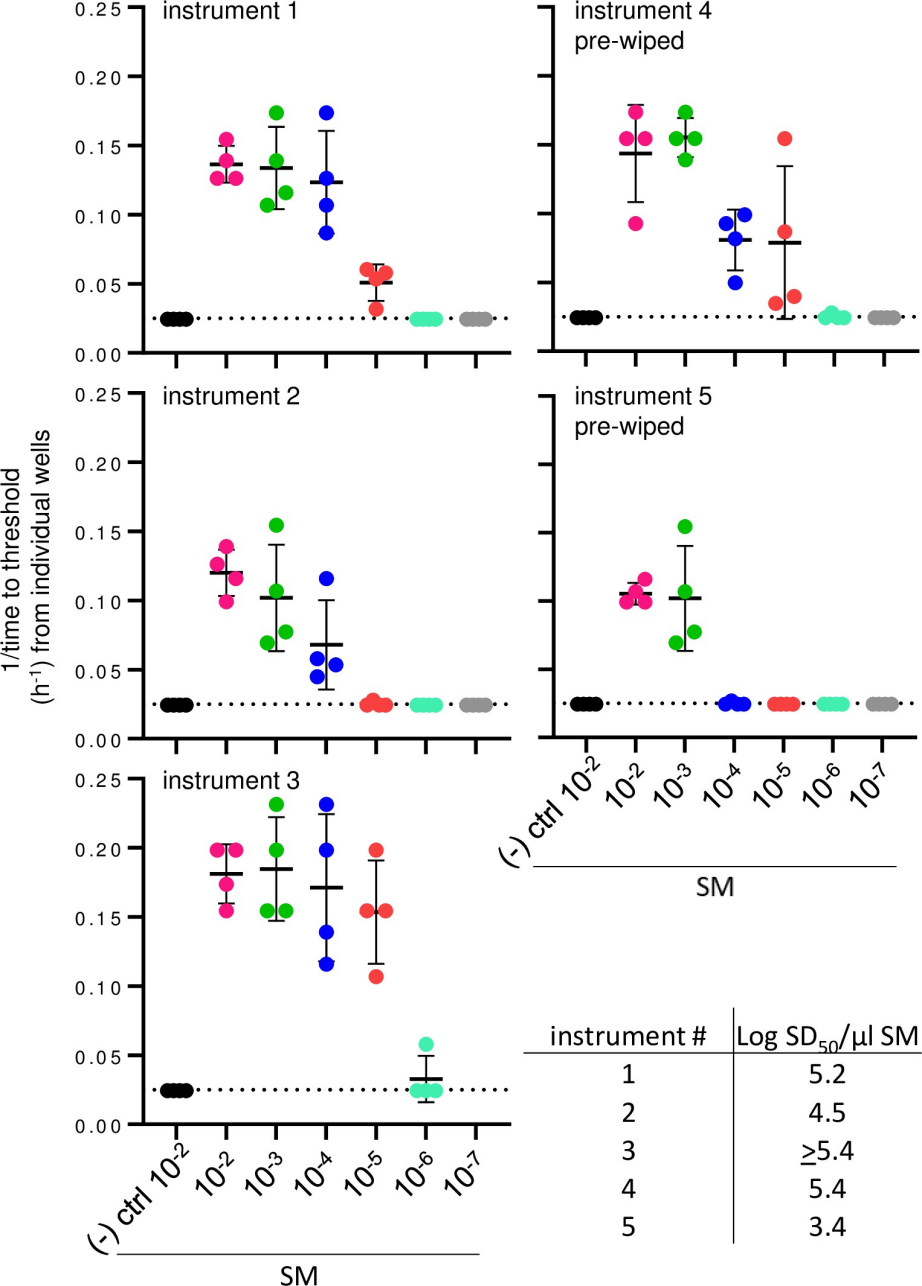
sfRT-QuIC detection of prions bound to stainless-steel surgical instruments after use on sCJD-infected Tg66 humanized mice. sfRT-QuIC data from serially diluted SM collected from forceps and scissors contaminated with brain tissue during routine surgery represented as 1/time to threshold for each reaction versus SM dilution as otherwise marked as in Fig 4B and 4D.

We then tested sfRT-QuIC in a more clinically relevant scenario. We used surgical instruments (forceps and scissors) that had been used to cut brain tissue from sCJD-infected transgenic mice expressing only human PrP (Tg66) [46], dry wiped with gauze (n = 2) or not (n = 3), and then air-dried prior to the application of SM (Fig 2C). For three of the instruments, including a wiped scissor, SM dilutions of 10^−2^–10^−5^ were positive in all quadruplicate wells, while SMs from the other two instruments were uniformly positive out to 10^−3^ and 10^−4^ dilutions (Fig 7). In contrast, no sfRT-QuIC positivity was seen on instruments used to dissect uninfected Tg66 mice brain tissue (Fig 7). These findings indicated that multiple logs of prion seeding activity can be detected on surgical instruments that had been used during necropsy of sCJD infected animals.

### Effects of post-sampling SM storage and omission of recombinant PrP in SM

In the experiments described so far, we included recombinant PrP^Sen^ in the SM. However, to simplify the SM and make it more compatible with storage and shipping before and after sampling but prior to RT-QuIC analysis (e.g., to allow transfer to a centralized assay facility), we also tested collections using SM without recombinant PrP^Sen^ and various storage conditions of the SM. 10% sCJD, CWD, and classical scrapie brain homogenates (2 μl) were applied to stainless steel, allowed to dry, and exposed to SM that either contained or lacked recombinant PrP^Sen^ (S1 Fig). SM lacking recombinant PrP^Sen^ included the final concentration of SDS that would have otherwise come from the seed diluent. We then stored the SMs for 0–7 d at 4°C, or 7 d at -20°C. We then thawed the samples, and added recombinant PrP^Sen^ to the SMs that initially lacked it, and performed end-point dilution RT-QuIC analysis of the SMs. The estimated seed concentrations (log SD50/mg original brain equivalent) recovered in the SMs were within a log of each other regardless of the presence or absence of recombinant PrP, or the storage time prior to RT-QuIC (S1 Fig A). Occasionally a positive replicate well was observed in the negative control samples, most notably with the uninfected cervid brain (S1 Fig B); however, as described in the methods these sporadic apparent false-positive wells were unrelated to the storage conditions and did not change the outcome of the assay as these samples did not meet criteria to be considered positive. Our results show that sensitive surface detection can be achieved using SM that does not contain recombinant PrP^Sen^ and that SM can be stored under a variety of conditions prior to adding the recombinant PrP^Sen^ for delayed RT-QuIC testing.

### Effect of pre-rinsing on detection of sAD, DLB, and sCJD-associated seeds on stainless-steel surface

We then tested effects of a water wash on detection of seeds from brain homogenates dried onto stainless steel (S2 Fig). In all cases, water prewash had little effect on the seed concentrations recovered in SM aliquots applied to the dried spots. Overall, our results showed that single transient water rinse did little to reduce the levels of α-syn, tau or prion seed contamination in brain homogenate dried onto stainless-steel.

### Effect of standard sterilization treatment of stainless-steel wires on sAD, DLB and Parkinson’s disease (PD)-associated seeds

**Fig 8:**
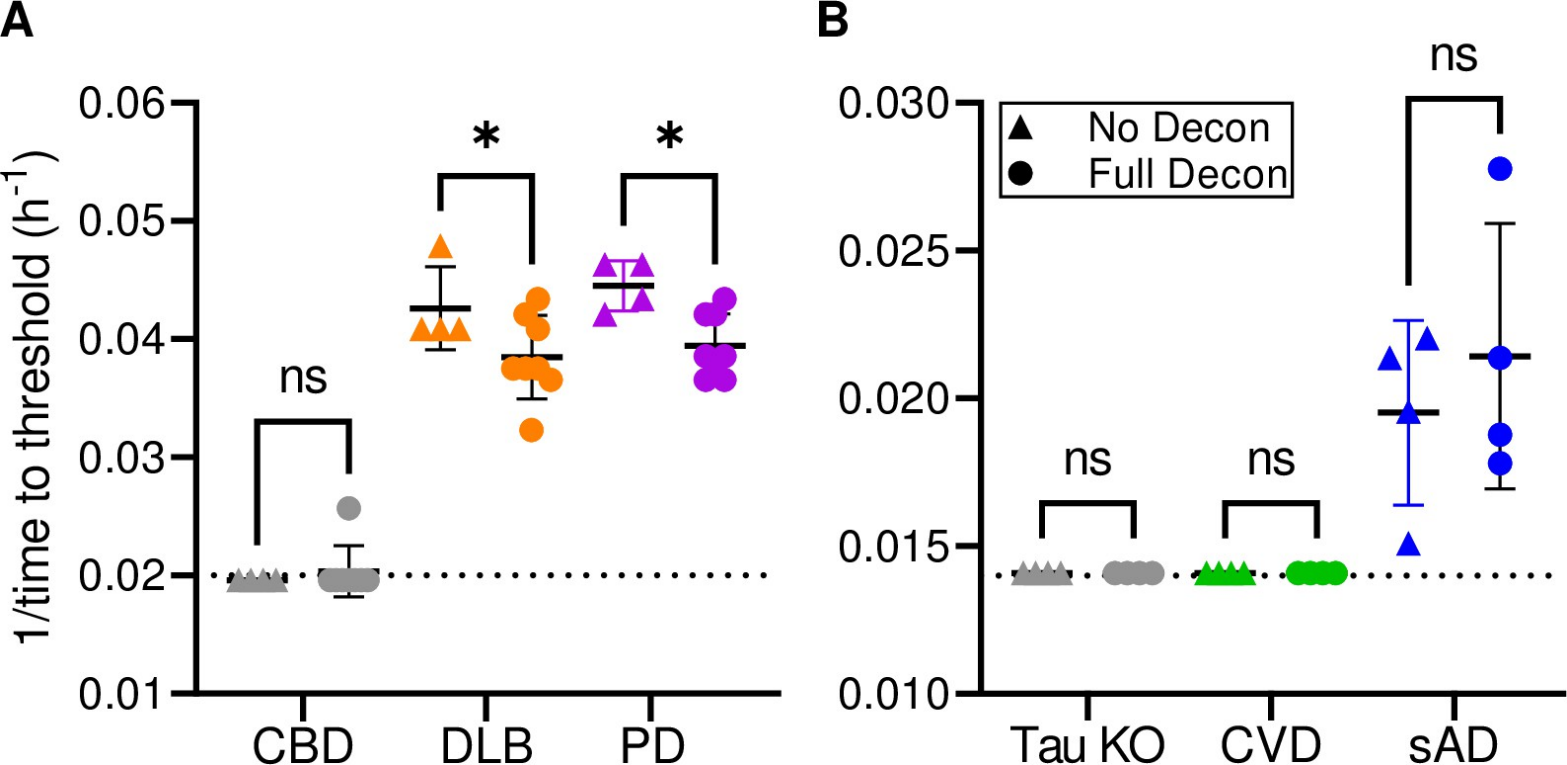
Effect of sterilization treatment of wires contaminated with sAD, DLB, and Parkinson’s disease (PD)-associated seeds. BH (10% w/v)-treated stainless steel wires tested by α-syn RT-QuICR for DLB, PD, as well as a CBD non-synucleinopathy control (**Panel A**) or K12 Tau RT-QuIC for sAD, as well as tau KO and CVD non-tauopathy controls (**Panel B**). Data points show 1/time to threshold values for untreated (triangles) and treated (circles) wires. Other markings as in Fig 4B and 4D. Asterisk (*) denotes a statistically significant difference (p<0.05) as analyzed by One-Way ANOVA; ns means non-significant.

Iatrogenic transmission of human prion diseases is well documented [22] and has led to the implementation of precautions and extreme decontamination protocols when, for example, surgical procedures have been performed on suspected CJD patients [6,24]. However, if prion disease is not suspected, reusable surgical instruments are typically subjected to a milder decontamination process that includes: 1) wiping/rinsing to minimize tissue debris, 2) treatments with disinfectant and/or enzymes (e.g., Medline Enzymatic Surgical Instrument Detergent and Presoak tested below), 3) rinsing with water and 4) sterilization by autoclaving (e.g.: 270°F/132°F for 5 min under pre-vacuum). To test if such a decontamination protocol would eliminate surface-bound α-syn or tau seeding activity, we assayed stainless steel wires contaminated with brain homogenate from patients with DLB or PD with α-syn RT-QuICR or sAD with the K12 Tau RT-QuIC (Fig 8). We detected wire-bound DLB- and PD-associated seeding activity even after the decontamination protocol described in Methods. While all reactions with the wires subjected to the decontamination protocol still showed positive seeding activity, slower reaction kinetics suggests a moderate reduction in seeding activity. We observed a similar phenomenon, albeit with more variable amplification kinetics, when testing Alzheimer’s disease contaminated stainless steel wires by K12 Tau RT-QuIC (Fig 8). We note that because the wires could not be fully submerged in reaction mix in 384 well plates, (with which the K12 assay was developed), we used 96 well plates for these experiments. It is possible that this atypical set-up led to sub-optimal sensitivity. Nevertheless, our results provide evidence that, as is known to be the case for PrP prions [47–49], standard cleaning and sterilization protocols may not inactivate proteopathic seeds bound to stainless-steel.

## Discussion

Persistent prion contamination of surfaces is a well-demonstrated risk in environmental, medical and wildlife settings. More recently, evidence of experimental transmission of some types of disease-associated α-synuclein, tau and β-amyloid seeds [6,50–55] have prompted concerns about surface contamination by these types of seeds as well. sfRT-QuIC provides a new method of detecting certain pathological protein seeds on surfaces that are too large to immerse in an RT-QuIC reaction well. In the case of prion sfRT-QuIC at least, SM that has been exposed test surfaces can be stored for at least a week at -20°C before RT-QuIC analysis. The ability to use SM without rPrP^Sen^ substrates should minimize the chances of false-positive assays due to spontaneous nucleation. This feature may make sfRT-QuIC testing more robust and accessible to those who lack on site testing capabilities.

Quantitative comparison of sfRT-QuIC assays to our standard solution RT-QuIC assays showed that not all of the seeding activity that was initially deposited on solid surfaces was detected by sfRT-QuIC, but the difference was less than ~0.8 log. This loss is not surprising and is likely due to some combination of an inability of transient SM exposure to recover bound seeds and inefficiency of permanently bound seeds in inducing nascent fibrilization of the substrate monomers while exposed transiently to SM (when the SM contains the RT-QuIC substrate). However, even if sfRT-QuIC detection of seeds in dried deposits is not quite as sensitive as solution RT-QuIC analysis of the corresponding initial brain homogenate, it still can provide highly sensitive detection of proteopathic seed contamination of solid surfaces.

Effective prion decontamination of non-disposable surgical instruments can be important to avoid iatrogenic transmission of human and possibly animal prions. In a recent study [56], Hervé et al reported that, despite the implementation of national guidelines to address prion contamination concerns in the UK, microscopic levels of proteins, which could include prions, remain on many reprocessed instruments after sterilization. As we have shown by testing humanized mouse prion contaminated surgical tools, sfRT-QuIC has the potential of being used to screen such surgical instruments for the presence of prion seeds. Furthermore, we have shown that α-syn and tau seeding activity can survive a commonly-used decontamination procedure for surgical instruments. Previous studies have also similarly reported that synuclein, tau and amyloid-beta can be difficult to remove from steel wires [57–59]. Interestingly, each of these proteins appear to have their own specific sensitivities to different, more aggressive types of inactivation.

The epidemiological risks of iatrogenic transmission of synucleinopathies, tauopathies, and β-amyloidoses appear to be low and remain under investigation and debate. However, some lines of evidence suggest that such transmissions can occur, albeit very rarely via invasive procedures [55,60–62]. The ability to sensitively monitor potentially infectious pathologic seeds on the surfaces of surgical instruments and medical devices such as endoscopes as reported here and by Yuan et al [19] should be helpful in assessing and mitigating such risks.

In this study we mainly focused on sfRT-QuIC detection on large smooth surfaces such as stainless steel or acrylic, which may be found in hospital or butchering and food processing settings. More uneven and porous surfaces such as wood or concrete have been shown to retain prions more tenaciously [26]. Further testing is required to discern the utility of sfRT-QuIC in those scenarios.

The studies by Yuan et al [18,19] have described a complementary RT-QuIC-based prion surface detection assay to ours for CWD and rodent prion strains. Another recent study described a PMCA- and swabbing-based method for detecting CWD prions on naturally exposed surfaces [40]. Here, we have extended the surface RT-detection approach to a wider variety of human and animal prion strains, as well as to pathologic seeds associated with α-synucleinopathies and tauopathies. While our respective assays show similar sensitivities, sfRT-QuIC provides a simpler protocol that does not require swabbing, sonication, or vacuum concentration steps. Also, in contrast to the Yuan protocol, the SM in sfRT-QuIC is not proteinase K-digested, which suggests that the sfRT-QuIC might be better suited to the detections of both protease-sensitive and resistant forms of prions or other types of seeds. With these improvements, sfRT-QuIC provides a simple and effective, high throughput method for sensitive detection of proteopathic seed contamination on common surfaces. The easy and cost-effective seed collection protocol paired with the compatibility with storing and shipping SM samples to RT-QuIC capable facilities around the world should facilitate its implementation.

Our current study presents an initial panel of prototypic sfRT-QuIC seed amplification assays. However, given the plethora of diseases that are now understood to involve the accumulation of various types of self-propagating amyloid fibrils [3, 5, 63–66] it will be important to expand the panels of sfRT-QuIC assays to the many proteopathic aggregates that may contaminate solid surfaces under certain circumstances.

## Materials and methods

### Ethics statement

In the US, human brain tissue was collected postmortem after having obtained consent from the legal next of kin according to applicable legal codes. In Italy, autopsy for CJD diagnosis is statutory, and consent from the legal next of kin was not needed. In all cases, the research teams had permission to provide a diagnosis and utilize nervous tissue for research purposes. Brains from cattle infected with BSE and non-infected control brains were obtained in accordance with the regulations outlined in Guide for the Care and Use of Laboratory Animals of the National Institute of Animal Health (NIAH) and Guidelines for Proper Conduct of Animal Experiments of the Science Council of Japan. Procedures involving animals were approved by the Institutional Animal Care and Use Committee at the NIAH (approval numbers 10–005, 11–008, and 13–005). CWD-infected brain tissues were collected by Dr. Elizabeth S. Williams (University of Wyoming) as part of surveillance (mule deer) or depopulation (elk) activities. Brain from an experimentally inoculated sheep was obtained under Washington State University IACUC Animal Subjects Approval Form #3811.

### Brain tissues and homogenate preparation

Brain tissue was collected at the terminal stage of disease from cattle experimentally inoculated intracerebrally with either the C-, L-, or H-BSE prion strain were obtained from the NIAH, National Agriculture and Food Research Organization (NARO), Japan [67–69]. Brain samples from 6 naturally CWD-infected wild mule deer collected by Elizabeth Williams and pooled prior to homogenization [70,71]. Dr. Williams also provided pooled brain samples from 6 naturally infected captive Rocky Mountain elk that were depopulated in South Dakota [70,71]. Frozen frontal cortex brain tissues of ovine, bovine, cervid and human origin were weighed to prepare a 10% w/v homogenate using 1mm zirconia beads (Biospec) and a bead beater (Bead Mill24; Fisher Scientific) in phosphate buffered saline (PBS) at pH 7.4. Homogenization was done at room temperature (RT) at max speed for 1 min. Homogenates were spun at 2000 xg for 2 min at 4°C and supernatant was recovered to make single use aliquots to be stored at -80°C.

### Stainless steel surface contamination procedures

Stainless steel plates (Amazon; stock #87183) were washed with deionized water, sprayed with 70% ethanol and air dried for approximately 1 hour prior to spotting with biological materials.

Contamination experiments were set up to create different scenarios. To assess the overall sensitivity of our system, serial dilutions of 10% brain homogenates in PBS (pH 7.4) supplemented with 1xN_2_ (Gibco) were spotted onto stainless steel plates (2μl per spot) and allowed to air dry at room temperature for 16–24 h.

Stainless steel surgical instruments used during procedures on transgenic Tg66 mice (un-inoculated or inoculated and symptomatic for sCJD; animal protocol: 2021-011-E) were either allowed to dry at RT for several weeks or lightly wiped right after use, then air dried.

### Contamination and sterilization treatments of stainless-steel wires

Stainless-steel wires were incubated in 50 μL of 10% DLB, sAD, CBD, CVD or mouse Tau KO brain homogenate at RT for 1h and 30 min with 1200rpm shaking. Wires were then washed in mQH_2_O, brushed using a small cervical brush and then incubated in a commercial detergent/enzymatic solution (Medline Enzymatic Surgical Instrument Detergent and Presoak—Enzymatic Presoak Cleaner, Dual Enzyme, MDS88000B9) for 5 min at RT with 600 rpm agitation. Wires were then rinsed 3 times with mQH_2_O, autoclaved for 5 min at 270°F and air dried overnight.

### Recombinant α-synuclein, Tau and prion protein purification

Recombinant PrP (rPrP^Sen^) was purified as previously described [29,72]. Briefly, *Escherichia coli*,(Rosetta 2, EMD Biosciences) with the vector for the PrP sequence (Syrian hamster residues 90 to 231 [GenBank accession number K02234] or bank vole residues 23 to 230; Methionine at residue 109 [GenBank accession number AF367624]) was grown in Luria broth (LB) medium with kanamycin and chloramphenicol. Protein expression was induced by Overnight Express autoinduction system 1 (Novagen). Recombinant PrP was isolated from inclusion bodies following their denaturation using Ni-nitrilotriacetic acid (NTA) superflow resin (Qiagen) with an ÄKTA fast protein liquid chromatographer (FPLC). PrP was refolded on the column by guanidine HCl reduction gradient and eluted with an imidazole gradient as described [29]. Next, the protein was dialyzed into 10 mM sodium phosphate buffer (pH 5.8). The final PrP concentration was determined by absorbance measured at 280 nm. After filtration (0.22-μm syringe filter [Fisher]), the rPrP^Sen^ was aliquoted and stored at −80°C.

The α-Syn sequence (K23Q mutation, Accession number NM_000345.3) was engineered using Q5 Site-Directed Mutagenesis (NEB) using the primers CCACACCCTGTTGGGTTTTCTCAG and CAGAAGCAGCAGGAAAGAC, as previously described [73] using a pET28 vector with an N-terminal His-tag (EMD Biosciences). The recombinant α-Syn was purified as previously described [72].

Purification of K12CFh (residues 244–275 and 306–400 of the full-length human tau sequence with an alanine mutation at residue 322 [74], thereby rendering it cysteine-free; K12CF) was done as described in [72].

### RT-QuIC assays

The prion RT-QuIC reaction mix contained 10 mM phosphate buffer (pH 7.4), 300 mM NaCl, 0.1 mg/ml rPrP^Sen^, 10 μM thioflavin T (ThT), 1 mM ethylenediaminetetraacetic acid tetrasodium salt (EDTA), and either 0.002 or 0.001% sodium dodecyl sulfate (SDS) for reactions set up with hamster rPrP^Sen^ 90–231 or bank vole 23–230, respectively [75]. The former conditions were used for 263K, sCJD, gCJD, and CWD prions detection, and the latter for vCJD, FFI, gCJD, scrapie, C-, H- and L-BSE prions detection. For in-solution assays, brain homogenates were serially diluted 1:10, as indicated, in PBS with 1X N2 and either 0.1 or 0.05% SDS (giving final concentrations of SDS as indicated above). For surface assays, the sampling media (SM) was the complete reaction mix with the addition of SDS at the final reaction concentration, but with or without rPrP^Sen^ as designated.

For in-solution experiments and surface experiments in which the sampling media was diluted (Fig 2B), 98 μl of reaction mix were loaded into a black 96-well plate with a clear bottom (Nunc) and seeded with 2 μl of BH or SM dilution for a final reaction volume of 100 μl. For surface experiments where dilutions of brain homogenate were spotted onto the surface (Fig 2A), 100 μl of the recovered SM were loaded directly into each well. All experiments were run with a minimum of 4 replicate wells. Plates were sealed (Nalgene Nunc International sealer) and incubated in a BMG FLUOstar Omega plate reader at either 50 or 42°C for the hamster or bank vole protein substrates, respectively. ThT fluorescence measurements (excitation, 450 ± 10 nm; emission, 480 ± 10 nm [bottom read]) were taken every 45 min for an incubation duration of 50hs, with cycles of 60 s of shaking (700 rpm, double-orbital) and 60 s of rest throughout the incubation.

The α-synuclein RT-QuICR reactions were done in black 96-well plates with a clear bottom (Nalgene Nunc International) with each well preloaded with six glass beads (0.8 mm in diameter, OPS Diagnostics). Quadruplicate reactions were seeded as described for the prion RT-QuIC. Each reaction mix was 98 μL of solution adjusted to give final reaction concentrations of 40 mM sodium phosphate buffer (from a stock solution of 0.5 M monobasic and 0.5 M dibasic sodium phosphate solutions mixed at a ratio of 1:19 for a pH 8.0), 170 mM NaCl, 0.1 mg/mL K23Q recombinant α-Syn (filtered through a 100 kD MWCO filter immediately prior to use), 10 μM thioflavin T (ThT). The plates were closed with a plate sealer film (Nalgene Nunc International) and incubated at 42°C in a BMG FLUOstar Omega plate reader. Plates were subjected to cycles of 1 min shaking (400 rpm double orbital) and 1 min rest for at least 48 h. ThT fluorescence measurements (450 +/− 10 nm excitation and 480 +/− 10 nm emission; bottom read) were taken every 45 min for 50 h with fluorimeter gain settings adjusted to maintain fluorescence responses within an unsaturated range (in most cases).

For the K12 RT-QuIC assay, BHs were serially diluted in 10-fold steps in a dilution buffer containing 0.53% tau-free mouse (KO; B6.129S4(Cg)-Mapttm1(EGFP)Klt/J from Jackson Laboratories) and 1x N-2 Supplement (Gibco) + 10 mM HEPES. The K12CFh tau fragmentK12CFh substrate was thawed and filtered through 100 kDa filters (Pall). Concentration was adjusted to 6.5 μM ~ 0.1 mg/mL K12CFh in a buffer containing 40 mM HEPES, pH 7.4, 400 mM NaF, 40 μM heparin, and 10 μM thioflavin T (ThT). This mix was thoroughly mixed in a polypropylene boat by gentle rocking for ~ 10 s and 48 μL or 98 μL mix was added to each well of a 384- (for BH and SM seeded experiments) or 96-well (for wire experiments) optically clear bottom plate (NUNC), respectively. In-solution and surface experiments were seeded as in the prion RT-QuIC except for having a final volume of 50 μl in a 384-well plate and 8 replicate reaction wells. For wire experiments in 96-well plates, steel wires were loaded into 4 replicate wells with a final reaction volume of 100 μL in each well. Plates were sealed with clear adhesive sealing tape and placed in an Omega FLUOStar plate reader pre-warmed to 42°C and subjected to rounds of 1 min shaking, 500 rpm, orbital, and 1 min rest, with ThT fluorescence reads (450 excitation, 480 emission) taken every 15 min for 50hs in the 384-well format and 70hs in the 96-well format.

For all three assays the fluorescence threshold for a positive reaction was calculated as 10% of the maximum value any well reached per experiment within the experiment’s cutoff time. The threshold was calculated individually for each well plate to account for differences across experiments and between fluorescent plate readers. For a sample to meet the criteria for being considered positive, at least 50% of the replicate wells (e.g., ≥2 of 4) had to be positive. Inverse time to thresholds were calculated for each reaction. Wells that did not cross fluorescence threshold within the time cutoff were calculated as 1 h past the time cutoff (e.g., 1/51 h). End-point dilution analyses were analyzed using the Spearman-Kärber calculation [76] to provide estimates of the concentrations of seeding activity units giving positive reactions in 50% of replicate reactions, i.e., the 50% “seeding doses” or SD50’s as previously described [29].

## Supporting information

S1 Fig Effects of post-sampling SM storage conditions and the omission of rPrP^sen^ on sfRT-QuIC analysis of prion-contaminated stainless steel. **Panel A**. LogSD_50_/mg of original brain tissue estimates derived from end-point dilution analyses (n = 1 per human and n = 2 per ovine and cervid) of SM collections from plates contaminated with sCJD, CWD, sheep scrapie and uninfected BH controls after the indicated post-sampling storage of SM. Orange symbols show data from tests using SM that contained rPrP^Sen^ substrate (as in the previously described experiments). Blue symbols show data from test in which rPrP^Sen^ was omitted from the SM during sampling and storage but was added just prior to the RT-QuIC reactions. **Panel B.** Negative control test results showing the % positive wells for sfRT-QuIC analyses of SM dilution series (n = 1 per human and n = 2 per ovine and cervid) from uninfected human, cervid or ovine brains. The mean (horizontal line) and standard deviations (vertical lines) are displayed for each type of brain. Dotted horizontal line indicates the threshold of % positive wells for a sample to be considered positive (see Methods).
(TIF)

S2 Fig Effect of water washing of stainless-steel surfaces on sfRT-QuIC detection of sCJD, DLB or sAD seeds. Mean +/- SD of logSD_50_/mg of original brain tissue (see Methods) estimated from end-point dilution analysis of sCJD (n = 2), DLB (n = 1) and sAD (n = 1) SMs collected before or after a water wash.
(TIF)

S1 Appendix Excel file of data used to compile figures. (XLSX)
