# Supplementary figures and images for: Sensitive detection of pathological seeds of α-synuclein, tau and prion protein on solid surfaces
Source: PLoS Pathog. 2024 Apr 19;20(4):e1012175. doi: 10.1371/journal.ppat.1012175 (PMC11062561; doi:10.1371/journal.ppat.1012175)

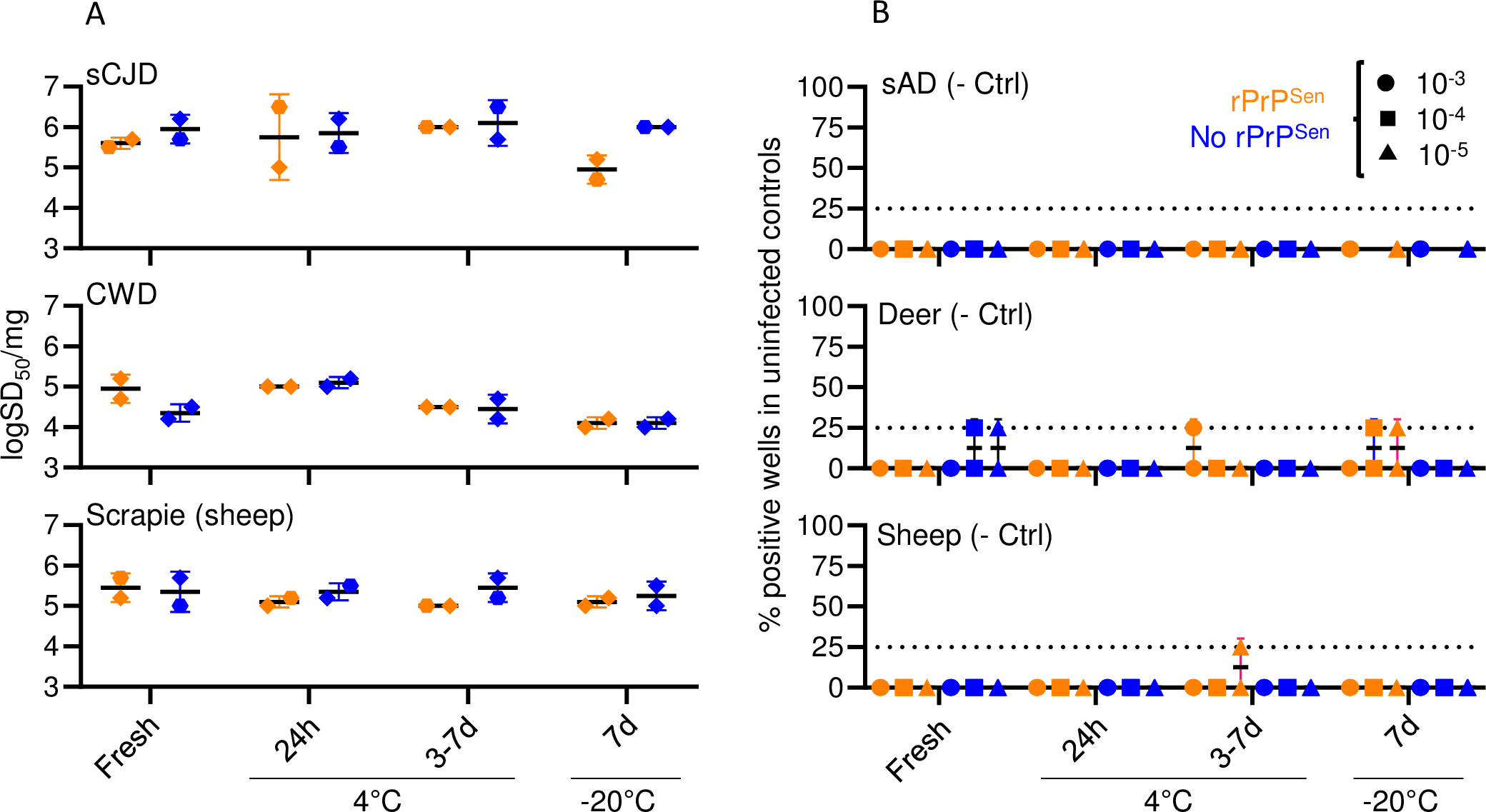

Supplement: S1 Fig — Panel A. LogSD50/mg of original brain tissue estimates derived from end-point dilution analyses (n = 1 per human and n = 2 per ovine and cervid) of SM collections from plates contaminated with sCJD, CWD, sheep scrapie and uninfected BH controls after the indicated post-sampling storage of SM. Orange symbols show data from tests using SM that contained rPrPSen substrate (as in the previously described experiments). Blue symbols show data from test in which rPrPSen was omitted from the SM during sampling and storage but was added just prior to the RT-QuIC reactions. Panel B. Negative control test results showing the % positive wells for sfRT-QuIC analyses of SM dilution series (n = 1 per human and n = 2 per ovine and cervid) from uninfected human, cervid or ovine brains. The mean (horizontal line) and standard deviations (vertical lines) are displayed for each type of brain. Dotted horizontal line indicates the threshold of % positive wells for a sample to be considered positive (see Methods). (TIF) [file ppat.1012175.s001.tif]

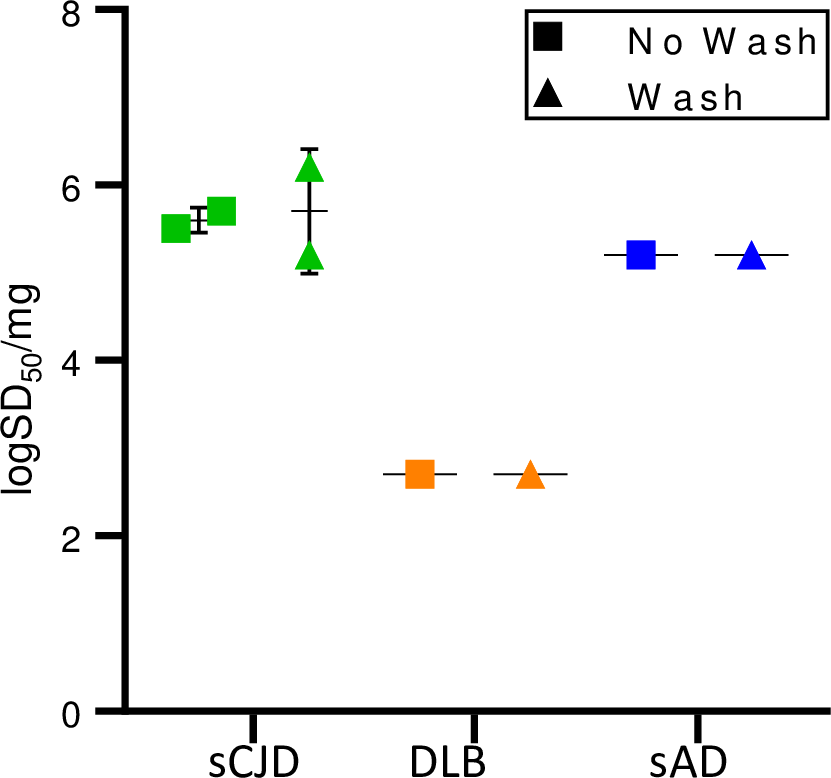

Supplement: S2 Fig — Mean +/- SD of logSD50/mg of original brain tissue (see Methods) estimated from end-point dilution analysis of sCJD (n = 2), DLB (n = 1) and sAD (n = 1) SMs collected before or after a water wash. (TIF) [file ppat.1012175.s002.tif]
